# Atlanta Society of Medicine

**Published:** 1899-07

**Authors:** 


### SOCIETY REPORTS.

### ATLANTA SOCIETY OF MEDICINE.

ATLANTA, GA., May 4, 1899.

*Regular Meeting of the Atlanta Society of Medicine.*

The meeting was called to order by the President, Dr. W. S. Goldsmith.

Dr. C. C. Geer read a paper on " General Anesthesia."

DISCUSSION.

*Dr. Kime:* In selecting a man to give the anesthetic I always get one in whom I have confidence. I have had some experience in not being confident of your man, and have found it a great disadvantage. I think the giving of an anesthetic should be as much of a specialty as other lines of special work. The man should be trained especially for that purpose, and it should not be left to incompetent hands. It seems to me that in the hospitals the nurses should be trained for this, so that in cases of emergency they could be relied upon for this work. Of course, there is no question that the anesthetist should devote all his attention to his work and none to the operation. Dr. Geer has expressed all these points very well indeed.

*Dr. Duncan:* I have listened attentively to this very excellent paper. I think he has given all the points in anesthesia and has given them well. For my part, I thank him for such a good paper.

*Dr. Hubbard:* I did not hear the first part of Dr. Geer's paper, but I was very much interested in what I did hear. I think, like Dr. Kime, that the man should make a study of anesthesia, if he is to give the anesthetic. I always prefer a man who has given it often, as I think the conditions can only be met by the training which comes from experience, and it is something which cannot be taught very well, but can only be acquired by experience.

There is one thing about which I differ with Dr. Geer, and that is the administration of nitroglycerin as a heart stimulant. As a prompt stimulant of the heart, I believe it is a good thing, but I think, however, that in the majority of cases we give too small doses, and instead of giving simply $\frac{1}{100}$ of a grain it can be given in $\frac{1}{50}$ grain doses with better results. I think that the failures with it are due to the fact that too small doses are given.

So far as telling the patient what to expect, and that they will not be entirely comfortable, I think the doctor is right there; for if the patient is told otherwise they soon find out differently, and then they lose confidence in the anesthetist, and this causes trouble.

*Dr. Hardon:* I think Dr. Geer has read one of the best papers I have ever heard read before this Society. I would take great pleasure in discussing it were it not for the fact that he has said everything necessary to be said. It is evident that Dr. Geer has profited by his experience, and probably more than most men. I have had a great many men to give the anesthetic for me, and the difference is very striking. I do not know of anything which is more embarrassing than to have a man to give the anesthetic who does not know anything about it. The man usually gives too little for fear he will give too much, but not with that rational fear with a knowledge of the dangers and how to avoid them. This fear is the proper feeling; but I believe that with inexperienced men that danger oftener arises from too little than from too much of the anesthetic. The man who is experienced will not give too much, and the inexperienced man will not give enough. I do not know anything which embarrasses a doctor more than to go to the country to do an operation and get a doctor there who has had no experience to give the anesthetic. He will not give enough, and while you are operating the patient will be struggling and the man will be sweating away and trembling for fear he will give too much when he is giving too little.

I think it is a good thing for an operator to always have one man to give the anesthetic for him. It contributes to the success of the operation in other ways besides the direct effect of the anesthetic. The surgeon cannot do the operation well if he has any doubt about his man.

I think this paper should be published. The profession should be educated up to the point of knowing how to give an anesthetic. It is surprising that the men in the cities know so little about giving this. It cannot be taught didactically but only by experience, but there are some men who do not profit by their experience. We make a mistake in allowing the physician who calls us into consultation to give the anesthetic. I think that we should insist upon having our own man, because, on account of the ignorance that prevails as to giving it, we cannot do the doctor justice and also the patient, and then danger arises.

*Dr. Elkin:* I have enjoyed this paper of Dr. Geer's very much indeed. It is a very practical paper, and has brought out all the points I hope to discuss. I not only enjoyed the paper, but have seen some of the doctor's practical work in this direction, and he has carried out to the letter in all cases the instructions he has given. We think it is of great importance to have a good anesthetizer; in fact the fear of the patient makes it a necessity. As a rule, the patient is not afraid of the operation but of the anesthetic, and they are more afraid the second time than before.

I have had some experience in this line, as I used to give the anesthetic quite frequently, and the more I gave it the more fear and dread I had of giving it. There was always more or less fear not that the patient would die, but it is necessary to be always on the alert.

The keynote of successful anesthesia is to gain the patient's confidence. If you get him to turn himself over to you completely he will go along all right and you will have very little trouble in administering the anesthetic. After you have gained your patient's confidence the thing to do is to look out for too much of the anesthetic; but, of course, if fully under the anesthetic there is less danger than if allowed to come from under its influence and to struggle, as the danger then is greater than of giving too much. This is a very important subject, and I am glad it was brought up to-night. Nothing gives more confidence in the operation than to have a good man to give the anesthetic. You cannot do the work well, as Dr. Hardon says, when you have to divide your attention between the operation and the anesthetic.

I cannot add anything to the practical points of Dr. Geer's paper. I thank him for it, and think it should be published.

*Dr. Butler:* I agree with Dr. Geer almost in full with everything he has said. I have given anesthetics a great many times and can appreciate the points, also from the standpoint of being operated upon.

There is one point, however, upon which we do not exactly agree, and that is in meeting the emergencies of the depressing effect. I believe that nitroglycerin is a good thing, because knowing its physiological effect and that it acts in about fifteen seconds and lasts about fifteen minutes. Following the hypodermic administration I have seen the cyanosed condition repeatedly disappear and rapid improvement in the condition of the patient. I have also had the same experience with the amyl nitrite. I think they are good things and like to rely upon them.

*Dr. Monroe Smith:* I have had no practical experience with anesthetics, only giving chloroform in obstetrical cases, but I noticed the point Dr. Geer mentioned of beginning the ether with plenty of air and to continue this way until almost anesthetized and then to push more rapidly. Last spring I was in New York and saw ether given a number of times daily. On one occasion Professor Phelps was waiting for a patient and made the remark that ether could be given almost as quickly as chloroform by giving plenty of air, but that they will struggle if you go too fast. In his final remarks, I would like for Dr. Geer to give his experience along that line.

*Dr. Goldsmith:* I can add nothing to the very valuable paper of Dr. Geer, except to thank him for presenting it, and can only say, with the others, that this knowledge should be diffused everywhere. Everybody seems to take it for granted that every one knows about giving anesthetics, but we all know of the ignorance which prevails in regard to its administration. I believe, with Dr. Hardon, in not allowing the consultant to give the anesthetic. On numerous occasions with Dr. Westmoreland, we were embarrassed in regard to this, and the man who operates can appreciate this point. I would prefer a third-course student who has given it a number of times, to a doctor who has been practicing a long time but not given an anesthetic.

*Dr. Geer* (closing the discussion): As to the use of nitroglycerin and amyl nitrite, many differ in regard to these and it is simply a matter of opinion.

The most important point is to gain the confidence of the patient, for the reason that if the patient is nervous and resists, it will take a longer time and a great deal more ether to anesthetize him, and this is especially true of smokers and drinkers.

As to slow anesthesia, I think that might be considered one way of winning the confidence of the patient. It is my experience that the slow is as quick a way as the other. When I first went into the Grady Hospital the method had been there to almost drown the patient with ether from the start and to have two negro men to hold the patient. The result was that the patient struggled and fought and it took an enormous amount of ether to get him under its influence. After I had been there a while the other method was adopted, and since then a stimulant is rarely needed and it does not take much time.

Dr. T. V. Hubbard read a paper upon "The Antiseptic and Eliminative Treatment of Typhoid Fever."

DISCUSSION.

*Dr. Kime:* This is a question which has been agitated for the last few years, and the profession is getting lined up on two sides; one, the antiseptic, and the other the let alone or expectant method. The latter claim that it is impossible to cut short an attack of typhoid fever. But the principal question that comes up and has been argued strongly against the antiseptic treatment is the possibility of disinfecting the alimentary canal. Those that favor the antiseptic method assert that the alimentary canal can be rendered aseptic; certainly sufficient evidence has been accumulated to show that it can be approximately disinfected. In my line I have argued along the line of elimination of the products of septic infection, and while we cannot render the uterus aseptic, yet we can accomplish this approximately through elimination by drainage. While we cannot render the alimentary canal aseptic, yet we can do it approximately.

Another important point in the treatment of typhoid fever is the effect of calomel upon the flow of bile in the alimentary canal. The men who do abdominal work and establish the elimination of bile through the alimentary canal have the best results. The flow of bile in the alimentary canal destroys the germs. In other words, where you have typhoid fever, the taking of the cathartic gets rid of the matter in the alimentary canal, toxic as well as otherwise.

A great many cases of typhoid fever can be relieved and cut short by proper treatment in the early stages. As to the plan of Dr. Woodbridge, I have never given the treatment on his plan. I have used my own plan, as every case cannot be like another. I first move the bowels and establish the functions of the different organs, and at the same time remember that the patient needs a nourishing diet. I think the man who fails to feed his patient fails in one of the essentials of the treatment. Also, the man who locks up the bowels and prays God that the bowels will not move is as dangerous as the man who moves them freely for two or three weeks. When the bowels are locked up you have a whole lot of coagulated lumpy matter coming in contact with the lacerated parts, and then you pour in oil and salts and get them moved in two or three days; but you have to use so much medicine and it is no wonder they die from hemorrhages. On the other hand, keep the canal clean by having the bowels moved and use the food to keep up the strength. Constipation also favors the development of bacteria and decomposition.

Build up the patient by good diet, and I also like the idea of building up the white blood cells with nucleins and protonucleins. The last, properly given, has the power of increasing the white blood cells, and iron, in the weak, debilitated condition, builds up the red cells. Also, for the nerves I use strychnine, and bitter tonics for the digestion. My experience along this line has given about as low mortality as you can get.

*Dr. Duncan:* There are two systems, as Dr. Kime mentions, for the treatment of typhoid fever, the doing nothing and the antiseptic treatment. I consider that doing nothing treatment can as justly be claimed to be malpractice as doing too much. I am well satisfied that doing nothing but watching the case and charging the party for your visits is malpractice just as well as doing something that is unnecessary. I am satisfied that typhoid fever needs treatment.

My plan is to use the best antiseptic remedies that can be had. For many years I used during the first week remedies per os and per rectum, giving antiseptics by mouth and flushing the bowels once a day with solution of salicylate of soda and boric acid. Some two years ago, just about the time the Woodbridge treatment was introduced here, I was called to see a case in the latter stages of the disease. The patient was a little girl, and she had several brothers, but she was the only one sick at that time. She was profoundly unconscious, and I could get her to take no medicine, and could not try the antiseptic treatment. She lived only a few days. A few days later I was called to see her brother. I put the patient on the Woodbridge treatment, beginning with Tablet No. 1, and continued on that treatment for a number of days, watching the effect, the bowels moving frequently, and improved every day. I could see that he was improving, although the bowels were moving so often. I continued the treatment and he got well. At the same time I had a man whom I put on the same treatment the same day, with the same frequency of the dose, and his bowels moved often, and he also got well. In the first family a younger brother was taken down with typhoid fever, as I believe, and I put him on the Woodbridge treatment and he did not have to lie in bed a whole day. I continued using the treatment and watching its effect while many of my brethren would not use it. I was willing to be called a fool if I could cure my patient. I did not lose another case that season after I adopted the treatment.

Recently I saw a little girl whom I thought had symptoms of typhoid fever; had a temperature of 102, tongue red around the edges and furred over the surface, but no tenderness over the abdomen. I went along watching the case until about the seventh or eighth day, and the typhoid eruption came out prominently over the abdomen, and I put her on the Woodbridge treatment. Her temperature had gradually gone up to about 104 or a little higher in the afternoon. She has been on the treatment now for about five days and her temperature has gradually gone down. Her temperature was 103 three days ago, 102 two days ago, 101 yesterday, and 100½ to-day. Her bowels have been loose but she is evidently doing well.

I do not agree with Dr. Hubbard that large doses of podophyllum are safe in typhoid fever. I do not like the effect of large doses. I like small doses, but I do not give continuously. I let them sleep when they can, but give when they are awake. I think the mortality of typhoid fever can be reduced with systematic treatment with antiseptics we now have, and I think the profession should be aroused to this point. Some of our leading men speak in favor of the do-nothing treatment of typhoid fever. I do not think they follow this, but they ought not to express themselves this way. The disease has killed millions, and will kill more, and we should do what we can to stay the disease.

*Dr. Hubbard* (closing the discussion): Gentlemen, I want to state that as regards the Woodbridge tablet, I have never used it, because I do not believe in disturbing the patient every fifteen minutes. At the same time, I cannot condemn it because I have never used it, and I do not think it fair for any one of us to condemn a thing without having had some experience with it. However, it seems to me that giving medicine every fifteen minutes would worry a patient. As to cutting the fever short or aborting it, I am satisfied that it is cut short, as it runs only about fifteen days. Some cases run their course, but the temperature after the first few days remains low, rarely ever going higher than 101, and the strength of the patient is preserved on account of this fact. Alcoholic stimulants are rarely indicated. I have given strychnine in some cases towards the last.

As to what extent the alimentary canal can be rendered aseptic, I cannot say, but I lay stress more on the eliminative part of the treatment. It is chiefly the stimulation of the eliminative function of the liver by mercury and podophyllum that I lay stress upon. If properly used, mercury is the drug for the disease, as in syphilis, but if improperly used it can do harm. As to guaiacol, we know its effect in tuberculosis, and I think we get the same effect in typhoid fever.

As I said before, I think it is the eliminative process which is of most value in the treatment. In appendicitis we know that purgatives will abort the disease, which is generally due to the colon bacillus, and it seems to me that the same principle would apply in typhoid fever. I do not claim that these drugs are the only ones that will give these results. Others may do it, but these are the ones I use. As to the size of the dose, I have never had any trouble. The indication is to eliminate as far as possible the source of the infection and the lethal product of the germs. It is not the germs so much as the toxins or product of the germs, but which cannot be separated from the germ itself.

Now, it is not what any man says that must finally settle this or any other treatment. It is the mortuary statistics that must settle as to whether this treatment is better than the cold bath. The Brann treatment does not meet the needs of the case. We have an infection on the inside, and pouring cold water on the surface does not remove the cause.

I do not ask any man to believe in this treatment because I said it, for I was a skeptic myself. I do not say that it will cure every case, but if you get the case in the first week, in the majority of cases, its course can be cut short. In any case you will have a speedy and more complete convalescence.

---

ATLANTA, GA., May 18, 1899.

*Regular Meeting of Atlanta Society of Medicine.*

The meeting was called to order by the president, Dr. W. S. Goldsmith.

Dr. Gilmer Robinson read a paper on "How can the Spread of Scarlet Fever and Diphtheria be prevented in Public Schools?"

DISCUSSION.

*Dr. Kime:* This is a question in which we are all interested, and especially physicians, and particularly those having children going to school. What stirred me up was the question of scarlet fever in our section for some time past. We have had more or less for months and years, but lately have had considerable excitement, and have had some spread of the disease, and I wonder we have not had more, from the way the matter has been handled. Being anxious about my own children, I went to the superintendent and had a conversation in regard to the cases in my section. As absence from school on account of sickness counts against the pupil, those who are anxious to have a perfect mark in attendance will attend school as long as they can possibly do so. One child went to school sick enough to have to rest upon a pillow. In speaking with the superintendent he said that he had had considerable difficulty in controlling the attendance of these children, and also that he had had difficulty with some physicians, and urged me to get better rules and regulations governing this condition. These facts brought my attention to the matter, and I asked the President to get some one to read a paper and get up a discussion.

I am informed as to how the disease spread within one certain family where a baby had an eruption and they thought it was scarlet fever, but a day or so later they decided that perhaps it was not scarlet fever. The children of the family continued to go to school, and a few days afterward a child had sore throat and this child went to school, but had to carry a pillow to rest upon. After this the third one was taken with the eruption, and in the mean time these children were attending school, and there is no law to control them. These were suspicious cases, and these are what we want to get at. This is what the superintendent wanted stress laid upon, that these children are allowed to go to school until the diagnosis has been made, and then very frequently the infection has been spread. About the time the third case was taken a consultation was called and a card was put up for diphtheria. I think four or five had the trouble, that is, according to hearsay. In less than three weeks the family has attended church with some of the children while the card is yet on the building. My wife was at church and sent her children home. This has occurred also in scarlet fever when the desquamation has not had time to cease. I do not think it safe for them to go out until three or four weeks after the height of the fever, and sometimes seven or eight weeks before they should be admitted into the general public. I do believe that any doctor that will cater to the public opinion sufficiently to allow a patient of scarlet fever to go away from home before the card is removed should not be allowed to practice, no matter who he is. He has no right to expose other people. My own children were exposed, and coming home to us this way we feel it keenly, and we should have very strict rules and regulations. There is scarlet fever on the Boulevard, and just below this is a case which is supposed to be, but there is no card, and on Jackson street there is ancther case. You see how it is spreading in our section with this laxness on the part of the authorities. It is the duty of somebody to take action in these cases. It seems to me that the Board of Health and the physicians are not working in harmony, and also that there is something lacking in regard to the rules and regulations, and I move that a committee of three from the Atlanta Society of Medicine, three from the Board of Health, and three from the Board of Education (including the Superintendent of Schools), be appointed as a committee to confer together to map out such rules and regulations as they think best for the control of the spread of contagious diseases in Atlanta.

*Dr. Stirling:* I think this is possibly the most important subject to bring up in a Society like this, because the prevention of disease is the highest form of medicine and this is developing more quickly than any other. I speak feelingly on this subject, because in England I was Officer of Health. It occurred to me when Dr. Robinson was reading his paper that a motion of this kind should be brought forward. I had it brought up three years ago when I was editor of the *Journal.* In England we are possibly more advanced in such matters, as the board of health is national there instead of being simply local. There they have a state board of health presided over by one chief officer with his subordinate men in the different cities and counties. He is in touch with the different officers of health throughout the country. These men are appointed by the local authorities in the various towns. In the larger towns a man is appointed with the understanding that his work is nothing else but looking after the work in that city. In the country he has possibly half a county, but his whole work is in looking after this part. Also, these men are all qualified for this task, as they have a special course of medicine in which they get special diplomas in chemistry, examination of water, etc., and therefore they are all qualified. The people of Atlanta should not value their health less than the people in other countries. It seems to me that this body, as representing the medical opinion of this city, should bring some such motion as this:

"That this city appoint some man especially qualified, whose whole duty should be to look after this matter, and when any infectious disease occurs, to visit that place as inspector and see that everything necessary is done to keep the death-rate down."

His duty should be to inspect all the various water supplies, see that all excreta are properly gotten rid of, inspect dairies, etc. If this is done Atlanta will be advanced, and there will be great improvement in the health of the city.

*Dr. Love:* The great trouble, as Major Slaton said to me, is the want of cooperation between the laity, the profession, and the municipal authorities. The Board of Education are the party of men who are supposed to look after the schools, but of course the work lies more in the hands of the superintendent. His duties are really more than he can look after. In this way the law is defective. The law is also defective in that it requires the physician to report only such cases as are infectious and contagious. As Dr. Robinson said, the danger from measles comes in the first stages, while in scarlet fever it is in the latter stages, and under the present rules and regulations there is no provision at all for a man to report a suspected case. Also, there are a great many people in Atlanta that never call a physician for a slight case of illness. As we know, they have cases whsch run their course without any physician being called. These may be infectious cases and the children are allowed to go to school. In scarlet fever the time of greatest contagion is in the desquamative stage. I am told by the superintendent that in a number of instances of scarlet fever that are reported by the physician to the Board of Health that the case is dismissed and the child is allowed to go back to school without one-fourth of the desquamative stage being through with. This is a fault, and the only way to get at the matter is according to

Dr. Kime's motion. At the same time, we might make it more far-reaching. We ought to get down to the groundwork and arouse the profession. There is where the trouble will be. I am sorry the majority of physicians are selfish and do not report these cases when they have them, and when they do report them, as soon as the fever passes the child is left to himself, and they give him some preparation to use during the desquamative stage and they report to the Board of Health that the child is turned loose upon the public. I think that he should make a visit in two, three, and four weeks and satisfy himself that all points of danger are past and that there is no danger of further trouble in the case. In talking with Major Slaton, he mentioned the importance of reporting suspected cases, and that the physician should not only keep that child at home but all others in the family. I told him that the trouble with this would be the cooperation of the parents of the children, because the rules were such that in keeping the child out for a certain time it counted against the child, and the parents would therefore object to keeping the children at home. He said that on this line he would assure the profession and the laity that suitable rules would be made for such cases so that the children would not be marked for such absences. If this were known the people would be more in favor of cooperating with the Board of Health. I have had parents tell me from day to day that the child would be marked for being out of school.

We should get together and form a committee and have men to go into this matter and sift it to the bottom, and take plenty of time, and when we form rules and regulations and have them adopted by the council, have them far-reaching enough to cover every point at issue. When we do this, and have laws to cover all ground, we will not only have the physicians but the people at large to co-operate with you.

As Dr. Robinson stated (with all due respect to the Board of Health), they are too lax in enforcing their laws, and then, too, they are not sufficient to cover all the ground. Until this is remedied we cannot hope to do anything in this matter. I think that if the profession would bring the subject before the other members of the profession that we meet, and get up some spirit and enthusiasm, we can, by co-operation with the Board of Health and council, pass such laws as are needed, and bring about more in a short time than any other way. I heartily favor the motion of Dr. Kime.

*Dr. Collins:* The inefficiency of the present laws of the Board of Health, and the evil that results from the term "membranous croup," which is misleading, was demonstrated to me not long ago. A teacher in the public schools came to me after school one Friday and told me that there was a case of membranous croup in a certain family, and that some of the children were still coming to school, and asked me what she should do about it. I told her that it could only be treated as diphtheria. The children came back to school on Monday and were sent home. The next day the child which had the membranous croup died. Those children had been going to school for a week after the case was known. Whether it was due to the carelessness of the physician I do not know, but this should be controlled by an inspector, or some such means.

*Dr. Jones:* I have been interested in this discussion, first as a member of the profession, and second as a member of the Board of Health.

A law back of which there is not a united public sentiment is worse than useless. This is represented in every-day life. You see it in lynchings. You see it in Atlanta. The laws in regard to contagious diseases might be improved, but they cannot be carried out if the physicians do not back them up. Now, in suspected cases, the physicians will not report these, and how are you going to make them report such cases? You cannot tell when a man will do this, and such a law would not be worth a row of pins. There is a difference between Atlanta and Boston and England. As the country gets older, and becomes educated up to the point, you can gradually make the laws more efficient and thorough. You let a committee go before the council and ask for a medical inspector, and they would laugh at you. Also, such a man should have tact of a high degree, and should possess first-class diagnostic skill, and he would have to devote his entire time to this work, for a knowledge among the laity that he visits scarlet fever and diphtheria would cause them to avoid him. Taking all this into consideration, such a man would have to be paid about $5,000, and the council would throw up their hands in holy horror, and if the committee received courteous attention they could congratulate themselves. Also, the profession would do the same way. Take the attendance to-night as an evidence. The Board of Health requires that these diseases be reported. It puts up a card and has to see after the proper isolation. The doctor is to report when this case is ready to be dismissed and when it should be fumigated. All this is done upon the certificate of the attending physician. Suppose you have an inspector and he says the case is not ready for dismission, then you bring up a controversy between the two. Therefore, unless you have a man of tact you will have a row right there.

What Dr. Love said about the public schools is another thing to contend with. I would be willing to do away with the public schools and thus do away with this difficulty. I am not in favor of public schools. The children are crowded at school, and if they should stay away for three or four weeks they are afraid they will get behind and cannot go on. I have fought this, but it does not have any effect. However, I keep them away as long as necessary. I have kept them away as long as eight weeks, and I have one family now which has been kept away for three weeks, and I do not intend for them to return this session.

Until we get the hearty co-operation of the profession back of us we cannot accomplish anything. The Board of Health has authority now, but cannot do anything; and if necessary we can get additional authority. There will be no trouble about this; but there is a great deal of trouble in enforcing it. To illustrate: Two or three weeks ago a man came to me and said his physician was so and so, and that he had a case of scarlet fever in the house, which was also occupied by a dressmaker, and the card was frightening away the dressmaker's patrons, and he had requested the doctor to allow him to tack the card on the back of the house instead of the front. That man was sent to the Board of Health by his physician to ask for such a thing. The man came to me and I was not afraid to tell him; this doctor would not do it, but pushed him off on me. This is simply an illustration of why it will not work.

Another thing is that some of these cases are never recognized. We have seen a clearly-marked case in the second child, and the first child was recognized only by the history given by the mother. Very fortunately we do not have these cases as bad in Atlanta, as it does not seem to be as malignant as in other cities.

Again, we have no pathologist employed by the city, and no cultures are made in diphtheria, as the city has no man to make cultures. We have a city chemist, paying him $50.00 per month, and two members of the Board of Health thought the honor was enough.

We want the profession to back us up, and the sentiments expressed here, if carried into the families, would be a great help. So far as Dr. Kime's resolution is concerned, I think it would be well to incorporate only members of this society in this committee, and leave off members of the Board of Health and of the Board of Education. Already there has been a great deal of trouble between the superintendent and the profession. The teachers have gone against the profession, and will turn down certificates of vaccination. If we accomplish anything it will be through the profession. We go to the legislature and they laugh at us. After ten years we managed to get three pieces of Boards of Examiners. The profession does not back us up; we have had trouble in getting them to register at the office of the Board of Health, as the majority of them kicked, and registered under protest, and some prominent members of the profession refused to report births and cases of typhoid fever. I do not think we will do anything by increased legislation, as we have enough laws. The trouble lies in getting the profession to carry out what we have.

*Dr. Benson:* Dr. Jones has mentioned about all I could say. Dr. Robinson's paper is important, but the great trouble is in getting the profession to back up the Board of Health. We cannot get them to do it. We know there must be many more diseases than are reported at this office, and we have tried through our inspectors to reach and find out these things, but it is impossible. We tried before the council to get a bacteriologist and cannot get it, but if we had the united profession behind us they would soon be educated up to the point of demanding it. This is a serious question in which we are interested, and all should be interested. The Board of Health wants the opinions of the profession and are glad to have them.

*Dr. Robinson* (closing the discussion): I have nothing more in particular to add. As Dr. Jones says, I think the Board of Health has sufficient laws to cover all these cases. It is largely a matter of carrying them out, as the Board of Health is unquestionably an arbitrary body and can do whatever they like, even to turning the council out of their chamber if necessary. I think the majority of the physicians are behind us and are willing to back up the Board, but, of course, there are others who are not. Now, with these laws, if they made an example of some one, the more prominent the better, they would have little or no trouble afterward.

As to this matter of inspector, it seems to me that Dr. Jones has the figures a little too high. Of course, the position needs a man with a great deal of tact. In diphtheria, if culture is made the negative result is sufficient to settle it. If culture is not made the inspector is to see that the throat is clear and that the nose is not running. In scarlet fever he is supposed to look between the toes and fingers and be sure that desquamation has entirely ceased. If he can show this to the physician then the physician cannot object to it. He is to use tact, but with a reasonable amount there is no chance for argument. This would do away with sending out children who are only half through the desquamative stage. If the physicians knew they would be looked after they would be very careful about this.

As to suspected cases, I quite agree with Dr. Jones, that it is impossible to get at it if the doctor keeps his mouth shut. The only thing we can do is to request a report in suspected cases. If a man is not sure, he has the Board of Health to fall back upon, and I would not hesitate to call upon the Board of Health in such a case. To simply say that you cannot do a thing seems to me is rather a weak argument. I would suggest that, as Dr. Jones says, the laws are complete enough, the committee try to formulate some methods by which we can reach the profession and get the profession at large to back up the Board of Health, and get the people interested by agitating the question in the newspapers.

*Dr. Love:* Pardon me for speaking again, but this is a matter in which I am deeply interested. There is no use in talking; we have got to do something. We have to have some starting point. Dr. Jones suggested that we have laws enough. I want to differ with him just a small bit. The only law is the one printed form which they send out to the physicians. It is true that on its face the law seems far-reaching, but a slight addition would mend matters considerably. For instance, if it was thoroughly understood that when a child comes to school sick that the teacher had the power to send for one of the city physicians and let him see whether the child should be sent home, and that the time the child loses would not be counted against it, then things would be in much better shape.

As to suspected cases, if there was a law governing this point, that the physician should keep suspected cases at home, they would do it. There is no question that we have to depend largely upon the profession, and if we do not start we will never accomplish anything.

The Board of Health should not be a political organization, but should be entirely of physicians, and it is not a place for a layman. There should be more physicians on the Board of Health.

*Dr. Stirling:* It seems to me that if we let the council know the opinion of the Society of Medicine in this matter that it will have some weight. Let them know what we want, and that some changes are desirable. Also bring the matter before the public. The estimate of having a man to look after this matter is too much. I will undertake to get a thoroughly competent man to do this for $2,500, and for less.

Of course, men object to making changes, but if the Board will uphold the physicians they will have no trouble after the first month or two. It will never be a proper health board until there is a man to look after this. I think the sentiment here should be taken and sent to the council and the papers, and the people will then know what the doctors think.

After discussing different motions, it was finally decided to send a committee of three to consult with the council in this matter.

The President appointed on this committee Drs. A. W. Stirling, R. R. Kime, and G. P. Robinson.

---

### CHARMING WOMEN.

"That the beauty of women, like that of men, should be determined from the standpoint of advancing maturity," says the *Pacific Record*, "cannot be disputed. It is absurd to claim that the ripe, rich beauty of forty is less attractive than the budding immaturity of sweet sixteen. Where women live in harmony with nature's laws each stage of life has its own charm. The physical beauty of women should last, growing more and more mellow until the end. The fullness of beauty does not reach its zenith under age of thirty-five or forty. Helen of Troy comes upon the stage at forty. Aspasia was thirty-six when married to Pericles, and she was a brilliant figure thirty years thereafter. Cleopatra was past thirty years when she met Antony. Diane de Poictiers was thirty-six when she won the heart of Henry II. The king was half her age, but his devotion never changed. Anne of Austria was thirty-eight when described as the most beautiful woman in Europe. Mme. de Maintenon was forty-three when united to Louis, and Catherine of Russia thirty-three when she seized the throne she occupied for thirty-five years.

"Mlle Mar was most beautiful at forty-five, and Mme. Récamier between the ages of thirty-five and fifty-five. The most lasting and intense passion is not inspired by two-decade beauties. The old saw about sweet sixteen is exploded by the truer knowledge that the highest beauty does not dwell in immaturity. For beauty does not mean alone the fashion of form and coloring as found in the waxen doll. The dew of youth and a complexion of roses are admirable for that period, but a woman's best and richest years are from twenty-six to forty."

